# The significance of microRNA deregulation in colorectal cancer development and the clinical uses as a diagnostic and prognostic biomarker and therapeutic agent

**DOI:** 10.1016/j.ncrna.2020.08.003

**Published:** 2020-08-23

**Authors:** Alireza Ahadi

**Affiliations:** Department of Medical Genetics, School of Medicine, Shahid Beheshti University of Medical Sciences, Tehran, Iran

**Keywords:** microRNA, Biomarker, Colorectal cancer, Diagnosis and prognosis, Signaling pathways

## Abstract

Colorectal cancer (CRC) is one of the most widely recognized and deadly malignancies worldwide. Although death rates have declined over the previous decade, mainly because of enhanced screening or potential treatment alternatives, CRC remains the third leading cause of cancer-related mortality globally, with an estimated incidence of over 1 million new cases and approximately 600 000 deaths estimated yearly. Therefore, many scientific efforts are put into the development of new diagnostic biomarkers for CRC. MicroRNAs (miRNAs), one of the epigenetics categories, have demonstrated significant roles in carcinogenesis and progression through regulating epithelial-mesenchymal transition (EMT), oncogenic signaling pathways, and metastasis. Dysregulation of miRNAs expression has been reported in many cancers, including CRC. The expression profile of miRNAs is reproducibly altered in CRC, and their expression patterns are associated with diagnosis, prognosis, and therapeutic outcomes in CRC. Recently, many studies were conducted on the dysregulation of miRNAs as a diagnostic and prognostic biomarker in CRC. Among them, some miRNAs, which include miR-21, miR-34 family, miR-155, miR-224, and miR-378, have been more studied in CRC with more prominent roles in diagnosis, prognosis, and therapy. In the present review, we summarized the latest information regarding the dysregulated miRNAs in CRC and the advantages of using miRNAs as a biomarker for CRC diagnosis, treatment, and their function in different signaling pathways involved in CRC progression. Moreover, we described the translation of miRNA research to potential therapeutic applications in the management of CRC in clinical settings.

## 1 Introduction

Colorectal cancer (CRC) is the third most frequently diagnosed cancer worldwide, with an annual incidence of 1.4 million new cases and 694 000 deaths [1]. About 15% of CRCs are diagnosed in metastatic stages (stage IV), with an average survival rate of 2.5 years. In the last decade, CRC incidence rates increased by 22%, and CRC death rates increased by 13% among adults aged less than 50 years in the USA [2]. However, the precise aetiologic factors of these onset cases have yet to be elucidated. According to recent studies, CRC develops from precancerous lesions; thus, early diagnosis can reduce incidence and mortality. Also, finding a potential diagnostic and prognostic biomarkers will help us assess tumor initiation, progression, and response to treatment [3,4].

MicroRNAs (miRNAs) are a family of endogenous, small nonprotein coding RNA molecules that conduct their suppressive functions by direct binding to the 3′‐untranslated regions (3′-UTR) of target mRNAs [5]. Approximately two-thirds of the protein-coding genes are known to be regulated by miRNAs [5]. MiRNAs participate in various biological functions such as apoptosis, cell development, and differentiation [6,7]. Because of their central role in tumorigenesis regulation, miRNAs have attracted a great deal of interest as potential therapeutic targets or disease biomarkers [8,9]. Dysregulated expression of miRNAs in human tumors is shown in several studies. In oncology, miRNAs are classified as oncogenes or tumor suppressors, depending on the function of their target genes [6]. Dysregulation of miRNA expression is related to the promotion of tumor mass growth, metastasis, increased malignancy of tumor cells. Moreover, the association between miRNAs expression and the risk of recurrence and response to the therapeutic regimen has been uncovered.

Therefore, miRNA profiling may be a novel tool for the diagnosis and prognosis of many types of tumors, including CRC. In this review, we summarized the differential expressions of miRNAs and their functions in CRC, and miRNA roles in CRC diagnosis, prognosis, and treatment.

## 2 Molecular pathogenesis of CRC

The suppressor pathway or pathway of chromosomal instability (CIN) was first proposed as the colorectal carcinogenesis [10]. The accumulation of mutations leads to oncogene activation such as Kirsten rat sarcoma (KRAS) and inactivation of TS genes such as Deleted in Colorectal Cancer (DCC), Total Protein-53 (TP-53), SMAD family member 4, Mothers against decapentaplegic homolog 4 (SMAD4), and Adenomatous polyposis coli (APC) [11]. Mutations in the genes MSH2, MSH3, MSH6, Exo1, PMS1, PSM2, MLH1, and MLH3 responsible for DNA repair during replication are associated with the second mechanism of colorectal carcinogenesis. Accumulation of errors in repetitive DNA fragments causes mutations in target genes [12]. The last pathway of aberrant hypermethylation was identified as a mechanism of gene function silencing in epigenetics [13]. Examples of these genes are the calcium voltage-gated channel subunit a1G (CACNA1G), the protein-coding gene, suppressor of cytokine signaling-1 (SOCS1), Runt-related transcription factor-3 (RUNX3), the induction of neuronal differentiation by the overexpression of NEUROG-1, and finally the insulin-like growth factor 2 (IGF2) [14].

## 3 A brief overview of microRNA

MiRNA represents the most studied non-coding RNAs, responsible for negative modulating of up to 60% of protein-coding gene expression [15]. Shortly, the biogenesis of miRNA starts in the nucleus, with the transcription of a long hairpin transcript (pri-miRNA) of hundreds or thousands of nucleotides. Further, by an enzymatic process coordinated by RNA polymerase III Drosha and DiGeorge syndrome critical region 8 (DGCR8), pri-miRNA is reduced to a smaller transcript of about 70 nucleotides, called pre-miRNA. After it is exported in the cytoplasm by nuclear receptor exportin, pre-miRNA is firstly reduced by Dicer complex to a mature miRNA duplex of about 22 nucleotides lengths and then to a single-stranded mature miRNA. Further, mature miRNA-loaded AGO2 and RNA-induced silencing complex (RISC) will function as a guide to target specific mRNA transcripts by sequence complementarity, usually in the 3′-UTR, leading to translational repression or mRNA degradation.

## 4 Aberrant miRNAs expression in CRC initiation and progression

### 4.1 miRNA expression on CRC proliferation

Aberrant expression of miRNAs and their roles in various biological processes have been observed to be associated with colorectal carcinogenesis. There is overwhelming evidence supporting a mechanistic role of miRNAs in various processes inside the cell, such as metastasis, cell proliferation, and apoptosis, which are considered a hallmark of CRC. During the last decade, scientific studies have been investigated the functional role of aberrant miRNAs expression profile in CRC proliferation.

MiR-143 with a tumor suppressive activity was identified in proliferation, and its expression is substantially reduced in CRC. Also, down-regulation of miR-143 can frequently precede APC gene mutations; thus, down-regulation of this miRNA is essential for the proliferation of CRC. Up-regulation of miR-21 targeted many genes, such as PDCD4, PTEN, transforming growth factor-beta receptor II (TGF-βR2), and cell division cycle 25A (CDC25A), that are involved in controlling CRC cell proliferation. Moreover, a recent study indicated that overexpression of miR-21 elevated cell proliferation and inhibited apoptosis against the treatment of chemotherapeutic agent Fluorouracil (5-FU) in an HT29 CRC cell [16,17]. Additionally, the silencing of miR-21 suppressed cell proliferation and restored the sensitivity of chemotherapy in HT-29 CRC cells.

MiR-155 by binding to the 3′-UTR of protein tyrosine phosphatase, receptor type J (PTPRJ) mRNA suppresses the expression of PTPRJ through miR-155/PTPRJ/AKT axis could affect the proliferation of CRC cells. MiR-143 and miR-145 can regulate cell growth and proliferation in vitro by targeting different oncogenic protein-coding genes [18,19]. MiR-143 by directly repressing the translation of KRAS [20] and DNMT3A [21] functions to suppress cell growth and proliferation [19]. MiR-148 induces cell proliferation and cell cycle progression in CRC by suppressing p55PIK. Moreover, the overexpression of miR-451 in CRC leads to decreased cell proliferation by targeting the oncogene macrophage migration factor (MIF) [22]. MiR-31 has been shown to increase CRC proliferation and tumorigenesis by directly binding to the 3′-UTR of RAS p21 GTPase activating protein 1 (RASA1) transcripts. Additionally, a recent study has shown that miR-29b suppress proliferation and induce apoptosis in CRC cells and mediate the inhibition of epithelial-mesenchymal transition (EMT) [23]. An overview of the studies investigating the role of miRNAs associated with CRC development is depicted in Table 1.

**Table 1:** miRNAs involved in CRC development.

miRNAs	Targets	Functions	References
miR-21	PDCD4	Invasion and metastasis promotion	[45]
miR-194	MAP4K4, AKT2	Proliferation, apoptosis, invasion, migration, cell cycle	[72,73]
miR-497	VEGFA	Inhibition of invasion and metastasis	[74]
miR-148b	CCK2R	Induction of cell proliferation	[75]
miR-409-3p	GAB1	Inhibition of tumor progression and metastasis	[76]
miR-92a	KLF4	Promotion of cell growth and migration	[77]
miR-100	RAP1B	Cell proliferation, invasion, apoptosis	[78]
miR-34a	E2F1, SIRT1, FMNL2, E2F5, SNHG7	Proliferation, invasiveness, metastasis, apoptosis,chemo-resistance	[[79], [80], [81]]
miR-638	SOX2	Cell invasion, migration, EMT	[82]
miR-320	FOXO4 and PDCD4	Inhibition of cell proliferation	[83]
miR-126	PI3K, VCAM-1, CXCR4, VEGFA,IRS1, RhoA	Proliferation, invasion, migration, cell cycle, angiogenesis, hematopoiesis	[84,85]
Let-7c	KRAS, MMP11 and PBX3	Metastasis induction	[86]
miR-503	calcium-sensing receptor	Induction of proliferation migration and invasion	[87]
miR-206	NOTCH3	Cell proliferation, migration, apoptosis, cell cycle arrest	[88]
miR-375	Bcl-2	Inhibition of tumor progression	[89]
miR-18a	K-Ras	Cell proliferation, anchorage-independent growth	[90]
miR-133a	FSCN1,LASP1	Cell proliferation, invasion, migration, tumor growth, intrahepatic and pulmonary metastasis, phosphorylation of ERK/MEK	[91,92]
miR-1246	CCNG2	Induction of cell growth and metastasis	[93]
MiR-330	CDC42	Proliferation	[94]
miR-320a	β-catenin,Rac1	Cell proliferation, migration, invasion, cell cycle arrest	[95]
miR-181b	RASSF1A	Proliferation and enhance cell survival	[96]
miR-124	STAT3	Cell proliferation, apoptosis, tumor growth, differentiation, prognosis	[97]
miR-144	GSPT1	Inhibition of proliferation and migration	[98]
miR-145	Fascin-1	Cell proliferation, invasion, tumor growth, pulmonary metastasis	[99]
miR-218	BMI-1	Cell proliferation, apoptosis, cell cycle arrest	[100]
miR-99b-5p	mTOR	Inhibition of metastasis formation	[101]
miR-429	Onecut2	Cell migration, invasion, EMT	[102]
miR-139	IGF-IR, NOTCH1	Cell proliferation, migration, invasion, apoptosis, tumor growth, cell cycle arrest	[103]

### 4.2 miRNAs affect the invasion in CRC

During CRC development, neoplastic cells may acquire the ability to invade or spread to distant organs through complex processes, including directional activation of proteolytic enzymes, EMT and translocation of cancer cells. During EMT, cancer cells undergo several processes that modify their phenotype, leading to cell motility, the acquisition of stemness properties, inhibition of apoptosis, and immunosuppression.

One of the critical miRNAs involved in the regulation of EMT-MET plasticity in CRC is miR-200. The overexpression of this miRNA in CRC cells contributed to MET through increased E-cadherin and reduced vimentin expression. miR-34a is another miRNA involve in EMT through snail1 as a target gene, which leads to induce EMT in CRC. In another relevant study, the expression levels of miR-155 promoting CRC cell invasion by regulating claudin-1 expression can act as a mediator of EMT. Moreover, miR-301 involves the regulation of invasion by targeting the downstream gene TGF-βR2 or NF-κB/STAT3 to promote tumorigenesis. MiR-29 family (miR-29a, miR-29b, and miR-29c) by regulating EMT are involved in the tumor progression. Specifically, the down-regulation of miR-29c has a vital role in CRC cell invasion via suppressing EMT in vitro. Also, miR-29b suppresses EMT and plays a vital role in cell invasion via negatively regulating the MAPK/ERK and PI3K/AKT pathways. Moreover, miR-126 within the 7th intron of epidermal growth factor-like domain 7 (EGFL7) could contribute to the progression of invasion and cell survival in CRC via inactivation of the oncogene signaling pathway.

### 4.3 miRNAs involve in metastasis

Metastasis is the final step during CRC progression. Recent studies implicate the components of miRNA-regulating networks in EMT with traits associated with metastasis formation in CRC. According to the recent evidence, miR-34a suppresses metastasis in CRC through EMT-regulating network in SNAIL/ZNF81 and IL6R/STAT3. MiR-20-5p and mir-224 can induce EMT and metastasis of CRC cells by negative regulation of SMAD4 as a mediator of the TGF-β pathway. Also, miR-34a inhibited CRC cell metastasis through the down-regulation of formin-like 2 (FMNL2) and E2F transcription factor 5 (E2F5) expressions. Additionally, miR-200c has a critical role in the regulation of EMT and metastatic behavior in CRC via the negative regulation of the target genes such as ETS proto-oncogene 1, transcription factor (ETS1), fms related tyrosine kinase 1 (FLT1) and zinc finger E-box binding homeobox 1 (ZEB1), which, in turn, regulates the EMT markers (E-cadherin and vimentin). Moreover, miR-224 can induce CRC tumor growth and metastasis by targeting SMAD4 [24]. Additionally, decrease the expression of miR-335, miR-132, and miR-192 can induce CRC metastasis via increasing expression of the ZEB2 target gene. Moreover, miR-126 inhibited the expression of vascular cell adhesion molecule-1 (VCAM-1), which led to metastasis in CRC. Another example is given by miR-200, whose increasing serum levels are significantly associated with CRC progression and metastasis.

## 5 miRNA involved in signaling pathways related to CRC

**Fig. 1:**
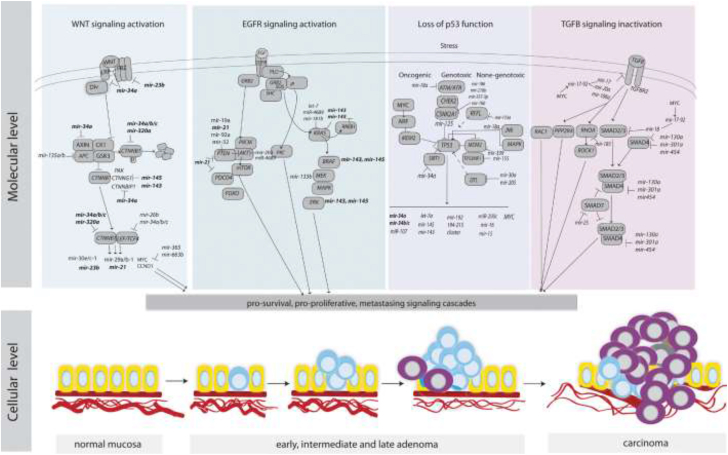
An overview of key signaling pathways in CRC and the regulation of their components by miRNAs.

One of the significant causes of CRC is the activation of driven genes in the oncogenic signaling pathways, such as TGF-β, Wnt, inflammatory signaling pathways, and Ras (Fig. 1). Also, these signaling pathways are regulated by miRNAs.

Recent studies indicated that miR-135a/b could regulate the Wnt signaling pathway by targeting APC the critical elements of the Wnt pathway, which leads to the repression of APC expression and induces of Wnt signaling pathway [25]. Also, the miR-34 family (miR-34a/b/c) can directly target Wnt ligands that interact with β-catenin [26], which leads to Wnt signaling repression [26].

Recent studies have reported that aberrant activation of the oncogenic EGFR pathway may occur due to TS-miRNAs loss of function. MiR-143 and miR-145 are the two most important TS in the EGFR pathway that decreases proliferation and migration by targeting KRAS and BRAF [27]. Most recent studies indicated that the crosstalk between the TGF-β signaling pathway and some miRNAs [28,29]. Several studies have shown that miR-20a, promotes CRC progression by facilitating CRC cell line migration, invasion and upregulating the expression of EMT markers, and further enhances the ability of TGF-β to drive cancer cell migration, invasion, and metastasis [30,31]. Similarly, miR-106 a/b also increases EMT and metastasis by targeting TGF-β receptor TGFBR2. MiR-20-5p and miR-224 by negative regulation of SMAD4 induce EMT, invasion, and metastasis of CRC cells [32,33].

One of the critical drivers of CRC is inflammatory signaling pathways, and miR-21 as a most recognized oncogene appears to be a key modulator of several pro-oncogenic and immunomodulatory factors, such as NF-κB, and MyD88, an adapter of Toll-like receptors (TLRs) needed for NF-κB activation by TLR ligands [[34], [35], [36]]. Similarly, miR-221 and miR-222 can activate NF-κB and STAT3 by indirectly modulating their protein stability through miR-221/222-mediated positive feedback loops to elevate the expression of STAT3 and RelA [37]. Therefore, the miR-21 family acts as a key modulator in inflammatory signaling pathways in which these miRNAs maintain a positive loop with the modulation factors PDCD4, NF-κB, and STAT3. More data about miRNAs involved in the signaling pathways in CRC developing and progression are presented in Table 2.

**Table 2:** The target genes of the dysregulated miRNAs and key signaling pathways contributing to CRC development.

miRNAs	Targets	Signaling Pathways	Oncogenic roles	Reference
miR-10b	PIK3CA, TGF-β, SM α-actin; TWIST-1, E-cadherin; KLF4; HOXD10, RhoC	PI3K/Akt/mTOR pathway, TGF-s signaling pathway	Tumorigenesis and metastasis; proliferation; vascular invasion, tumor differentiation and metastasis	[104,105]
miR-638	SOX2, TSPAN1	Suppressing of TP53 function	EMT, invasion, migration, proliferation	[82,106]
miR-574-5p	Qki 6/7	Wnt/β-catenin pathway	Proliferation, tumorigenesis differentiation, angiogenesis	[107]
miR-21	TGFBR2, CTNNB1, PIK3CA, ZFHX3, BRAF, SFRP1, ITGb4, PDCD4,PTEN, TIAM1, TIPM3, SPRY2, RECK, and Sec23A	Activation of Wnt/s-catenin pathway; TGF-s signaling pathway	Tumor progression, proliferation, EMT, metastasis; invasion, inhibition of apoptosis induction of stemness	[[108], [109], [110], [111]]
miR-217	MAPK1, KRAS, Raf-1	EGFR signaling pathway	Tumor growth, apoptosis	[112]
miR-137	CTNNB1, WNT3a	Wnt/β-catenin pathway	Cell cycle progression	[113]
miR-19a	KRAS, VEGFA	EGFR signaling pathway	Proliferation, angiogenesis	[114]
miR-96	RECK, TP53INP1, FOXO1, and FOXO3a	Activation of Wnt signaling pathway	Cell cycle progression, decreased apoptosis	[113]
miR-504	TP53	Suppressing of TP53 function	CRC progression	[115]
miR-106a	PTEN, PI3K, and AKT; *TGFBR2*	PTEN/PI3K/AKT signaling pathway; TGF-s signaling pathway	Tumorigenesis, inhibition of cell apoptosis and autophagy; increased migration, invasion and metastasis	[116]
miR-182	ST6GALNAC2, PI3K/AKT	EGFRsignaling pathway	Proliferation, invasion	[117]
miR-222	PTEN	PTEN signaling	Radio resistance, metastatic activity	[118]
miR-384	KRAS, CDC42	EGFR signaling pathway	Invasion, migratiuon, metastasis	[119]
miR-150-5p	TP53	Deficiency of TP53 function	Promotion of proliferation, cell cycle progress, invasion/migration, reduction of cell apoptosis	[120]
miR-146a	NUMB	Wnt/β-catenin pathway	Progression, stemness	[121]
miR-26b	PTEN, WNT5A	Induction of EGFR signaling pathway	EMT, proliferation, and metastasis	[122]
miR-181a	SRC kinase signaling inhibitor 1 (SRCIN1); WIF-1, E-cadherin, β-catenin, and vimentin	Promotion of SRC/VEGF signaling pathway; wnt/β-catenin signaling	Angiogenesis; cell motility, invasion, tumor growth and liver metastasis	[123,124]
miR-125a-3p	FUT5-FUT6	EGFR signaling pathway	Proliferation, migration, invasion, angiogenesis	[125]
miR-135b	TGFBR2	Inactivation of TGF-β signaling pathway	Progression, inhibiting of apoptosis	[126]
miR-92a	PTEN, SMAD2, SMAD4, and TGFBR2; KLF4; matrix metalloproteinase 2 and E-cadherin	PI3K/Akt pathway	Proliferation, EMT, invasion, venous invasion, and metastases, cell growth and migration.	[127,128]
miR-494	APC	wnt/β-catenin signaling	Proliferation, tumorigenesis	[129]
miR-224	SMAD4	Inactivation of TGF-β signaling pathway	Invasion, metastasis	[33]
miR-185	MYC, CCND1	Wnt/β-catenin pathway	Proliferation, progression	[130]

## 6 miRNA could act as diagnosis and prognosis biomarker in CRC

MiRNAs with high stability in many types of biological samples have become an outstanding candidate for discovering new cancer biomarkers [38,39]. In recent years, several reports have demonstrated that miRNAs can be a potential biomarker for the diagnosis and prognosis of CRC [40,41]. For example, a panel of six miRNAs, including miR-21, let-7g, miR-31, miR-92a, miR-181b, and miR-203 are reliable biomarkers in CRC diagnosis with over 80% specificity and sensitivity [42]. Another study conducted by Hibner et al. [43] shows the panel of 7 miRNAs (let-7a, miR-1229, miR-1246, miR-150, miR-21, miR-223, and miR-23a) act as a potential biomarker for CRC diagnosis and prognosis with high sensitivity and specificity [44]. One of the most identified oncogenic miRNAs, which are highly expressed in CRC is miR-21, which has been linked to carcinogenic processes [[45], [46], [47]]. These findings highlight the importance of miR-21 as a molecular biomarker [48,49]. In the study that the overexpression of miR-21 was found in serum samples from CRC patients [50], the authors proposed a three-miRNAs panel (miR-21, miR-19a-3p, and miR-425-5p) for diagnosis of CRC with the high sensitivity and specificity in CRC serum samples (0.875 and 0.744, respectively) with an area under the ROC curve of 0.88 [51]. Additionally, another study indicated that miR-21 as an early diagnostic biomarker for CRC with a sensitivity and specificity of 0.77 and 0.84, respectively [52], with an AUC of 0.81. An interesting study was conducted by Pan et al. [52], in which the expression level of 30 miRNAs in plasma samples was analyzed using qRT-PCR. These authors showed that analysis of plasma expression levels of five miRNAs, such as miR-15b, miR-17, miR-21, miR-26b, and miR-145, together with carcinoembryonic antigen (CEA), can improve the diagnostic accuracy of CRC (AUC = 0.85 in the training cohort, AUC = 0.818 in the validation cohort).

The increased expression levels of miR-155 in CRC tissues compared to normal samples showed by Zhang et al. [53] after analyzing clinical samples of patients with CRC. Moreover, based on the recent study conducted by Lv et al. [54], they discovered that there is no change in serum miR-155 expression level between controls and stage Ⅰ CRC patients after measuring the serum specimens of CRC patients compared to healthy controls. However, the up-regulation of miR-155 in stages Ⅱ-Ⅳ patients was found. Thus, miR-155 cannot be used as an early diagnostic biomarker in serum [54]. Moreover, a recent study has shown that overexpression of miR-155 in CRC patients shows poor overall survival (OS) and disease-free survival (DFS), this study proposed that miR-155 has independent prognostic values for OS and DFS in CRC patients [55]. Thus, miR-155 might serve as a new tumor biomarker in the clinicopathological diagnosis and prognostic assessment in CRC.

Moreover, another example of miRNA in plasma with the highest predictive capability in CRC is miR-378 [56]. According to the association between the miR-378 decrease and increased tumor volume, metastasis, and short OS of CRC patients and the tumor suppressor role of this miRNA, all of the above suggested that miR-378 could serve as a biomarker to predict the outcome of CRC [57]. The list of some crucial miRNAs as diagnostic and prognostic biomarkers in CRC is summarized in Table 3, Table 4.

**Table 3:** Dysregulation of miRNAs as diagnostic and prognostic biomarkers of CRC.

miRNAs	Sources of miRNA	Expression	Sensitivity	Specificity	Biomarker	References
miR-19a	Serum	up-regulation	66.7%	63.9%	Prognostic	[131]
miR-144	Serum	up-regulation	74%	87%	Diagnostic	[132]
miR-106a	plasma	up-regulation	62.3%	68.2%	Diagnostic	[133]
miR-21	Serum	up-regulation	82.8%	90.6%	Diagnostic and Prognostic	[47]
miR-601	Plasma	down-regulation	69.2%	72.4%	Diagnostic	[134]
miR-18a	Serum	up-regulation	61%	69%	Diagnostic	[135]
miR-92	Plasma	up-regulation	89%	70%	Diagnostic	[136]
miR-183	Plasma	up-regulation	73.7%	88.5%	Diagnostic and Prognostic	[137]
miR-145	Serum	down-regulation	–	–	Diagnostic and Prognostic	[138]
miR-23a-3p, miR-27a-3p, miR-142-5p, miR-376c-3p	Serum	up-regulation	89%	81%	Diagnostic	[139]
miR-92a	Plasma	up-regulation	71.6%	73.3%	Diagnostic	[136]
miR-451	Plasma	up-regulation	88.2%	100%	Diagnostic	[140]
miR-139-3p, miR-431	Plasma	up-regulation	91%	57%	Diagnostic and Prognostic	[141]
miR-96	Plasma	up-regulation	65.4%	73.3%	Prognostic	[142]
miR-7, miR-93	Plasma	down-regulation	91%	88%	Diagnostic	[143]
miR-17-3p	Plasma	up-regulation	64%	70%	Diagnostic and Prognostic	[138]
miR-760	plasma	down-regulation	80%	72.4%	Diagnostic	[143]
miR-375	Plasma	down-regulation	76.92%	64.63%	Diagnostic and Prognostic	[144]
miR-422a	Serum	down-regulation	–	–	Prognostic	[145]
miR-1290	Serum	up-regulation	70.01%	91.2%	Diagnostic	[146]
miR-221	plasma	up-regulation	86%	41%	Diagnostic	[135]

**Table 4:** Identifying prognostic values of miRNA for CRC via univariate and multivariate analysis.

miRNAs	Sources of miRNA	Expression	HR (95% CI), P value		Outcome	References
			Univariate analysis	Multivariate analysis		
miR-17-3p	Serum	up-regulation	2.72 (1.58–4.69), P < 0.0001	2.24 (1.28–3.92), P = 0.035	DFS	[138]
miR-96	Plasma	up-regulation	P = 0.002	2.27 (1.15–4.51), P = 0.019	OS	[142]
miR-23a-3p, miR-376c-3p	Serum	up-regulation	–	2.30 (1.44–3.66), P < 0.0004	OS	[139]
miR-106a	Serum	up-regulation	2.81 (1.64–4.80), P < 0.0001	3.02 (1.36–6.73), P = 0.007	DFS	[138]
miR-141	Plasma	up-regulation	3.61(1.96–6.65)	2.40 (1.18–4.86)	OS	[147]
miR-1290	Serum	up-regulation	3.43 (1.83–6.67)	4.51 (1.23–23.69), P = 0.0096	OS	[146]

## 7 miRNA can act as a therapeutic target in the CRC

One of the significant troubles for CRC treatment is the acquired chemotherapy resistance. As the miRNAs are involved in cancer progression, they can be considered therapeutic targets [58]. Different tools may be used for the miRNA inhibition, such as the miRNA sponges, antisense oligonucleotides, or molecule inhibitors. Downregulation of miR-211 sponge was indicated against the TUSC7 in the CRC tissues compared to the normal ones. Furthermore, The survival rate of high-expression miR-211 in patients is superior to those with low expression [59]. Moreover, one of the most critical issues in miRNAs therapeutics is the use of miRNA as a replacement therapy via inhibition of miRNA function through anti-miRs and miRNA mimics [60].

MiRNAs can be silenced by anti-miRs, antagomiRs, locked nucleic acids (LNAs), or miRNA sponges. A recent study showed that LNA-*anti*-miR-21 inhibited cell growth and invasiveness in LS174T CRC cells, suggesting the therapeutic potential of LNA-*anti*-miR-21 in CRC [61]. Similar studies showed the action by the anti-miRs against the miR-20a [62], miR-21 [63], miR-95 [64], miR-675 [65], and miR-31 [66] in the CRC cell lines. One of the most exciting strategies in cell culture of CRC by targeting overexpressed oncogenic miRNAs is that miRNAs bind to the RISC complexes, leading to blocking the interaction of miRNAs with their endogenous mRNA targets. Specific inhibition of miR-20a, miR-21, miR-95, and miR-675 has been achieved in human CRC cell lines to inhibit cell proliferation and induce apoptosis [62,65,67]. Also, miRNAs can act as predictive biomarkers for therapeutic response in CRC due to the high tissue specificity and stability.

One of the main components of therapy for CRC treatment and its proven effect on survival in CRC patients is 5-FU [68]. The low expression levels of miR-34 were observed in 5-FU-resistant CRC DLD-1 cells, and this miRNA was investigated as a recurrence biomarker due to sensitizing cells to 5-FU treatment inhibits cell growth [69]. Additionally, miR-21 by down-regulation of MutS homolog 2 (MSH2) confer resistance to 5-FU chemotherapy; conversely, the up-regulation of this miRNA suppresses apoptosis induced by 5-FU and G2/M arrest [63].

Downregulation of miR-34a was shown to mediate resistance to 5-FU in the CRC cell line, reversing the resistance by downregulating Sirt1 and E2F3 via ectopic expression miR-34a [69]. More importantly, treatment with miR-21 and miR-30d antagonists were sensitized hypoxic and resistant CRC cells to 5-FU [69]. Moreover, according to the study conducted on the expression profiles of miRNAs in the plasma from 24 CRC patients before and after four cycles of 5-FU/oxaliplatin treatment. The significant upregulation of (miR-106a, miR-484, and miR-130b) in non-responders before treatment was observed. According to the recent experiments, overexpression of miR-153 increased CRC resistance to oxaliplatin both in vitro and in vivo [70].The upregulation of miR-409-3p inhibited cell autophagic activity and enhanced the sensitivity to oxaliplatin, abrogated by the restoration of beclin-1, suggesting that miR-409-3p sensitized CRC to oxaliplatin by inhibiting beclin-1-mediated autophagy [71]. Several studies have focused on miRNAs regulatory roles in the induction of chemo-resistance and their involvement in treatment success (Table 5).

**Table 5:** miRNAs and their expression pattern in response to drugs in CRC.

miRNAs	Treatment regimen	Expression	Ref.
miR-21	5-FU	High	[46]
miR-214	5-Fu	Low	[148]
miR-143	Oxaliplatin	Low	[149]
miR-126	oxaliplatin	Low	[150]
miR-10b	5-Fu	High	[151]
miR-519c	5-FU	Low	[152]
miR-129	5-Fu	High	[153]
miR-625-3p	oxaliplatin	High	[154]
miR-148a	5-FU	Low	[155]
miR-106a, miR-130b, miR-484	5-FU, oxaliplatin	High	[156]
miR-143	Oxaliplatin	Low	[157]
miR-215, miR-190b, miR-29b-2	5-FU	High	[158]
miR-625-3p	Oxaliplatin	High	[154]
miR-320e	5-FU, oxaliplatin	High	[159]
miR-150	5-FU	Low	[68]
miR-143	5-Fu	Low	[160]
miR-203	Oxaliplatin	High	[161]
miR-494	5-Fu	Low	[68]
miR-1914	oxaliplatin	Low	[162]
miR-34a	5-Fu	Low	[69]

In sum, there are a limited number of studies have been conducted in miRNA-based therapy; thus, there may be a long way for the first miRNA-based therapy for CRC in the future.

## 8 Discussion

**Fig. 2:**
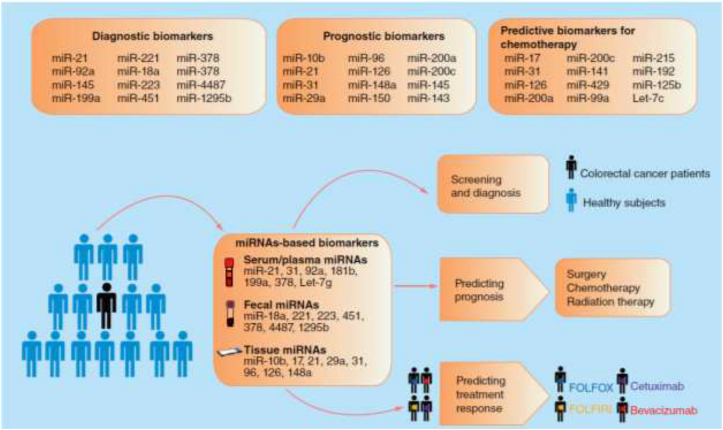
The overview of the clinical application of miRNAs in colorectal cancer.

Driving and modulation of the progression in CRC may be occurred by dysregulation of miRNAs. By understanding the regulatory roles of miRNAs in CRC initiation and progression, we can find new insight into finding novel diagnostic and prognostic tools for CRC screening and personalized therapy. The clinical perspective of miRNAs and the importance as a diagnostic and prognostic biomarkers for CRC are summarized in Fig. 2.

**Fig. 3:**
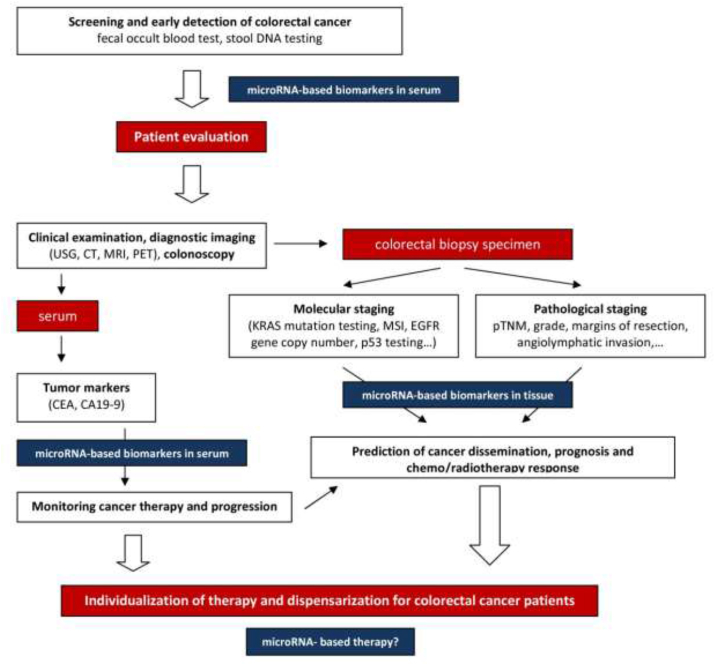
The potential usage of miRNAs in the clinical management of the colorectal cancer patients.

Recent studies indicated that due to the insufficient specificity and sensitivity using the expression profiles of a single miRNA as a diagnostic or prognostic biomarker of CRC, it is not most effective. It may be helpful to use the miRNAs signature together and conventional biomarkers, such as CEA, to increase the sensitivity and specificity. Many researchers are currently investigating miRNA panels as CRC biomarkers, which appears to be a more promising strategy than single miRNA tests. The development of panels containing many miRNA biomarkers seems essential and may enable more accurate diagnoses and prognoses of CRC in the future. Moreover, there are many challenges in the development use of miRNAs-based therapeutics for CRC. The potential use of miRNAs in the clinical management of CRC patients is summarized in Fig. 3. One of the main challenges in the therapeutic of CRC is the identification of miRNAs that affect CRC and determine how to achieve effective delivery without causing undesirable side effects. For example, one of the adverse effects of replacement therapy is the initial clinical experience with miR-34; thus, the correction of miRNA dysregulation is a promising therapeutic approach for CRC treatment.

In conclusion, despite the numerous studies of miRNAs in CRC have been conducted, but the roles and functions of many individual miRNAs in CRC remain poorly understood. Thus, the integrated analysis of multiple miRNA targets for a given miRNA, and the integrated bioinformatic analysis of mRNAs, proteins, copy number variants, and mutations, are strongly needed.

## Declaration of competing interest

The author declares that they have no conflict of interest.
